# A Second Endolysin Gene Is Fully Embedded In-Frame with the *lysA* Gene of Mycobacteriophage Ms6

**DOI:** 10.1371/journal.pone.0020515

**Published:** 2011-06-09

**Authors:** Maria João Catalão, Catarina Milho, Filipa Gil, José Moniz-Pereira, Madalena Pimentel

**Affiliations:** Centro de Patogénese Molecular, Unidade dos Retrovírus e Infecções Associadas, Faculdade de Farmácia, Universidade de Lisboa, Lisboa, Portugal; Instituto Nacional de Câncer, Brazil

## Abstract

Mycobacteriophages are dsDNA viruses that infect mycobacterial hosts. The mycobacteriophage Ms6 accomplishes lysis by producing two cell wall hydrolytic enzymes, Lysin A (LysA) that possesses a central peptidoglycan recognition protein (PGRP) super-family conserved domain with the amidase catalytic site, that cleaves the amide bond between the *N*-acetylmuramic acid and L-alanine residues in the oligopeptide crosslinking chains of the peptidoglycan and Lysin B (LysB) a mycolylarabinogalactan esterase that hydrolyzes the mycolic acids from the mycolyl-arabinogalactan-peptidoglycan complex. Examination of the endolysin (*lysA*) DNA sequence revealed the existence of an embedded gene (*lysA*_241_) encoded in the same reading frame and preceded by a consensus ribosome-binding site. In the present work we show that, even though *lysA* is essential for Ms6 viability, phage mutants that express only the longer (Lysin_384_) or the shorter (Lysin_241_) endolysin are viable, but defective in the normal timing, progression and completion of host cell lysis. In addition, both endolysins have peptidoglycan hydrolase activity and demonstrated broad growth inhibition activity against various Gram-positive bacteria and mycobacteria.

## Introduction

At the end of the replication cycle, bacteriophages must exit the host cell and disperse their newly formed progeny to infect new cells. The main barrier to host lysis is the peptidoglycan, a strong and stable structure that allows the bacterial envelope to withstand internal osmotic pressure [1]. With the exception of filamentous phages that as a result of their unique morphology and morphogenesis can extrude through the envelope without fatal consequences for the host, all other phages must either degrade or otherwise compromise the peptidoglycan to cause lysis [2], [3]. Most of the tailed double-stranded DNA (dsDNA) phages achieve the proper time for lysis by the consecutive use of two lysis proteins – holin and endolysin. Holins are small hydrophobic membrane proteins that during the late phase of phage development progressively accumulate in the cytoplasmic membrane of the host and while the proton-motive force is maintained assemble into oligomers and rafts of intrinsic stability [2], [4]. At a precise time programmed into its primary structure and upon a specific trigger event (holin effector concentration and partial depolarization of the membrane), the holin suddenly causes disruption of the membrane with non-specific hole formation and collapse of the membrane potential which sets the time of lysis by allowing the destruction of the cell wall by the released or activated phage encoded muralytic enzymes, the endolysins [5], [6]. The term endolysin is used to describe the dsDNA bacteriophage-encoded peptidoglycan hydrolases, which are synthesized in phage-infected cells at the end of the multiplication cycle. They are characterized by the ability to directly target bonds in the peptidoglycan layer of the bacterial cell wall; the result of this activity is degradation of the rigid murein layer and release of newly assembled virions by way of lysis [7], [8]. Endolysins distribute between five major functional types; i) *N*-acetylmuramidases (lysozymes); ii) endo-β-*N*-acetylglucosaminidases, which all hydrolyze the β-1, 4 glycosidic bonds in the murein; iii) transglycosylases, which attack the same bonds but form a cyclic 1, 6 anhydro-*N*-acetylmuramic acid product; iv) *N*-acetyl-muramoyl-L-alanine amidases, which hydrolyze the amide bond between the *N*-acetylmuramic acid and L-alanine residues in the oligopeptide crosslinking chains; v) endopeptidases, which attack the peptide bonds in the same chains. In phages infecting Gram-positive bacteria, the most common architecture includes two clearly separated functional domains: the N-terminal domain that generally harbours the enzymatic activity, whereas the cell wall binding domain located at the C-terminal region directs the enzymes to their substrates and may restrain the enzyme lytic action to a particular type of cell wall [3], [7]. Nonetheless, at least four bifunctional lysins have also been reported, consisting of an N-terminal and central catalytic domain with different specificity and a C-terminal substrate-binding domain; examples are the endolysins encoded by *Streptococcus agalactiae* bacteriophage B30 (muramidase and endopeptidase) [9], *Staphylococcus aureus* phage φ11 (endopeptidase and amidase) [10], *Streptococcus agalactiae* phage NCTC 11261 (endopeptidase and muramidase) [11], and *Staphylococcus warneri* M phage φWMY (endopeptidase and amidase) [12]. Most known endolysins lack signal peptide (SP) sequences and depend entirely on the cognate holins for release to the peptidoglycan. However, endolysins containing N-terminal secretory signals have already been described. The endolysin of *Oenococcus oeni* phage fOg44 is endowed with a bona-fide SP that is processed by the leader peptidase during infection and is exported by the *sec* machinery [13]. A survey of orthologous endolysins from other phages of Gram-positive hosts suggested that some of these have N-terminal sequences resembling secretory signals, although in every case an adjacent holin gene was also present [6], [13]. In addition, the endolysin of *Lactobacillus plantarum* phage φg1e has been reported as being exported in *E. coli* by the Sec machinery [14]. Particularly remarkable cases are the endolysins of *E. coli* phages P1 and 21, which feature an N-terminal signal-arrest-release (SAR) sequence that allows the enzyme to be exported to the membrane where it is arrested, and to be released as a soluble active enzyme in the periplasm [15]–[17].

The mycobacteriophage Ms6 is a temperate bacteriophage with an unusual lytic cassette: in addition to the endolysin-holin lysis system, encoded by genes *lysA* (*gp2*) and *gp4/gp5*, respectively, the Ms6 lytic cassette comprises two additional lysis proteins encoded by genes *gp1* and *gp3* (*lysB*) [18], [19]. The *lysB* gene has been previously characterized: it encodes an enzyme with lipolytic activity that hydrolyzes the mycolic acids from the mycolyl-arabinogalactan-peptidoglycan complex [20], [21] acting at a later stage of infection to facilitate lysis by compromising the integrity of the mycobacterial outer membrane linkage to the arabinogalactan-peptidoglycan layer [22]. The Ms6 *lysA* gene was shown to encode a 384 amino acid polypeptide (LysA) with significant similarity to some bacteriophage encoded lysins with *N*-acetylmuramoyl-L-alanine amidase activity [18]. Several types of cell wall hydrolases seem to be produced by mycobacteriophages. While phages Ms6 and TM4 encode enzymes with an amid-2 type domain [23], others such as D29 and Bxb1 employ hydrolases with lysozyme-like activity to bring about host cell lysis. Mycobacteriophage endolysins containing SP or SAR domains that allow secretion of the endolysin into the periplasmic space have not yet been described. Interestingly, however, our group has recently identified the product of *gp1* gene as a chaperone-like protein that specifically interacts with the N-terminal region of the Ms6 endolysin. Gp1 is involved in the endolysin translocation across the cytoplasmic membrane independently of the holin function and is required for efficient phage release [24]. During an attempt to purify LysA as a C-terminal histidine-tagged fusion product, we detected the synthesis of two proteins, rather than a single polypeptide. Further examination of *lysA* (*lysA*_384_) nucleotide sequence revealed a second possible gene (*lysA*_241_) in the same reading frame and preceded by a potential ribosome-binding site (RBS). Here, we report studies directed at dissecting the precise role of the *lysA*-encoded gene products during *Mycobacterium smegmatis* infection by the mycobacteriophage Ms6. In addition, the lytic activity spectrum of both proteins was also examined in both Gram-positive and Gram-negative bacteria and also in mycobacteria regarding the potential application of mycobacteriophage lysins.

## Materials and Methods

### Bacterial strains, phages, plasmids and culture conditions

Bacterial strains, phages and plasmids used throughout this study are listed in Table 1. *E. coli* strains were grown at 37°C, in Luria-Bertani (LB) broth or agar supplemented with 100 µg ml^−1^ ampicillin or 30 µg ml^−1^ kanamycin, when appropriate. *M. smegmatis* recombinant strains were grown at 37°C in 7H9 medium (Difco) supplemented with 0.05% Tween 80, with shaking or Middlebrook 7H10 (Difco), containing 15 µg ml^−1^ kanamycin. For induced conditions 0.2% succinate and 0.2% acetamide were also added to media.

**Table 1:** Strains, bacteriophages, plasmids used in this study.

Strains, bacteriophages or plasmids	Description	Reference or source
Bacteria		
*Escherichia coli*		
JM109	*recA1 endA1 gyr96 thi hsdR17 supE44 relA1* Δ(*lac-proAB*) [F′ *traD36 proAB lacI* ^q^ *Z*ΔM15]	Stratagene
BL21 (DE3)	F^−^ *ompT hsdS_B_* (r_B_^−^ m_B_^−^) *gal dmc* (DE3)	Novagen
*Mycobacterium smegmatis*		
mc^2^155	High-transformation-efficiency mutant of *M. smegmatis* ATCC 607	[26]
Bacteriophages		
Ms6*_wt_*	Temperate bacteriophage from *M. smegmatis*	[70]
Ms6-LysAHis_6_	His_6_tag insertion at the 3′ end of Ms6 *lysA*	[24]
Ms6*_Δgp1_*-LysAHis_6_	His_6_tag insertion at the 3′ end of Ms6 *lysA* in Ms6*_Δgp1_*	This study
Ms6-Lysin_384_His_6_	GTG→TGG change in codon 144 of *lysA* in Ms6-LysAHis	This study
Ms6-Lysin_241_His_6_	Stop codon introduced in the *lysA* gene of Ms6-LysAHis_6_	This study
Plasmids		
pQE30	Expression vector; T5 promoter; Amp^r^	QIAGEN
pET29b(+)	Expression vector, T7 promoter; Kan^r^	Novagen
pJV53	Derivative of pLAM12 with Che9c *60* and *61* under control of the acetamidase promoter; Kan^r^	[71]
pMJC40	*lysA_24_*_1_ Ms6 cloned into pQE30	This study
pMJC41	l*ysA_384_* Ms6 cloned into pET29b(+)	This study
pMJC42	*lysA_241_* Ms6 cloned into pET29b(+)	This study
pMJC43	*lysA_384_*_ΔGTG_ cloned into pET29b(+)ΔRBS	This study

**Ms6 lysis genes Accession No. AF319619.**

### Plasmid construction

Unless otherwise indicated, DNA fragments obtained by PCR were amplified using Ms6 genomic DNA as template. DNA amplification, plasmid isolation and electrophoresis were carried out using standard techniques [25]. *E. coli* and *M. smegmatis* mc^2^155 cells were transformed as described previously [25], [26]. Restriction enzymes and T4 DNA ligase (New England Biolabs) were used according to the supplier's recommendations. All oligonucleotides were purchased from Thermo Scientific and are listed in Table S1.

In order to construct plasmids pMJC40 and pMJC42, *lysA_241_* was amplified using primers *lysA_241_*fwd-1/p*lysA*-c3 or *lysA_241_*fwd-2/p*lysA*-c3 and the resulting DNA fragments were introduced into the BamHI/HindIII sites of vector pQE30, allowing a fusion to a hexahistidine tag at the N-terminus, or pET29b allowing a C-terminal hexahistidine tag fusion, respectively. To obtain plasmid pMJC41, the DNA fragment containing *lysA*, was amplified by PCR with primers gp2A/p*lysA*-c3 and cloned into BamHI/HindIII sites of pET29b fused to a C-terminal His_6_tag. Plasmid pMJC43 was constructed by PCR amplifying *lysA*_384_ (*lysA*) lacking the GTG start codon with primers p*lysA*ΔGTGfwd/p*lysA*-c3 and cloning the DNA fragment, after restriction with XbaI/HindIII which removed the vector translational signals (RBS and start codons), in pET29b. All constructs were validated by sequencing the insert nucleotide sequence.

### Construction of Ms6 mutant phages

Construction of Ms6 mutant phages was performed using the Bacteriophage Recombineering of Electroporated DNA (BRED) as described previously [24], [27]. Briefly, for Ms6 *lysA* deletion, a 100 bp oligonucleotide, PrΔ*lysA*, that has 50 bp of homology upstream and downstream of the region to be deleted was extended by PCR using two 75 bp extender primers, PrExtΔ*lysA*fwd/ PrExtΔ*lysA*rv, which have 25 bp of homology to the ends of the 100-mer and add additional 50 bp of homology on either end. The final 200 bp dsDNA product was purified using MinElute PCR Purification Kit (QIAGEN) and co-electroporated with Ms6*_wt_* genomic DNA into electrocompetent recombineering cells of *M. smegmatis* mc^2^155:pJV53. To abolish synthesis of Lysin_384_ and Lysin_241_ we designed two complementary 73 bp oligonucleotides (Pr*lysA*_TGA_HindIIIfwd/ Pr*lysA*_TGA_HindIIIrv) that introduce a stop codon and a HindIII restriction site downstream of the start codon of *lysA_384_*, or two complementary 86 bp oligonucleotides (Pr*lysA*_GTG→TGG_MscIfwd/ Pr*lysA*_GTG→TGG_MscIrv) that modify the *lysA_241_* GTG start codon (valine) to TGG (tryptophan) and introduce an MscI restriction site, respectively. Complementary oligonucleotides were co-transformed with Ms6-LysAHis_6_ genomic DNA into recombineering cells of *M. smegmatis* mc^2^155:pJV53. Cells were resuspended in 7H9 supplemented with 0.5% glucose and 1 mM of CaCl_2_, incubated at 37°C for 2 hours (prior to lysis) and plated as top agar lawns with *M. smegmatis* mc^2^155. Phage plaques were picked into 100 µl phage buffer (10 mM Tris-HCl, pH 7.5; 10 mM MgSO_4_; 68.5 mM NaCl; 1 mM CaCl_2_), eluted for two hours at room temperature and analysed by DADA-PCR [27] with primers Δ*lysA*DADA-PCR/*lysB*DADA-PCR to detect *lysA* deletion. To detect *lysA* TGA insertion and *lysA* GTG_144_→TGG change, phage plaques were analysed by PCR with primers PrP1fwd/*lysA*180bprv or gp2A/gp2B followed by HindIII or MscI restriction, respectively. Mixed primary plaques containing both mutant and wild-type DNA were eluted as described above, and serial dilutions were plated with *M. smegmatis*. Individual secondary plaques or lysates were screened by DADA-PCR for *lysA* deletion or by PCR and restriction with the same primers referred above to identify pure mutant phages. Construction of phage Ms6_Δ*gp1*_-LysAHis_6_ was done as described previously [24] using Ms6_Δ*gp1*_ genomic DNA.

### Lysin expression in *M. smegmatis*-infected cells

Examination of Lysin_384_ and Lysin_241_ synthesis in *M. smegmatis* was performed as previously described [13]. An exponentially growing culture of *M. smegmatis* mc^2^155 was infected with Ms6-LysAHis_6_, Ms6_Δ*gp1*_-LysAHis_6_, Ms6-Lysin_241_His_6_ or Ms6-Lysin_384_His_6_ at an approximate multiplicity of infection (MOI) of 10 and incubated at 37°C for 30 minutes. Ten-mililiter samples were withdrawn at 30-min intervals; cells were pelleted by centrifugation and frozen at −20°C. After thawing, cells were concentrated 100-fold in phosphate-buffered saline supplemented with 20 mg of lysozyme ml^−1^. After an incubation period at 37°C for 1 hour, 25 µl of 5× SDS-PAGE sample buffer were added followed by incubation at 100°C for 5 minutes to complete cell lysis. *M. smegmatis* extracts were analysed by Western blotting and lysin immunodetection was performed using horseradish-peroxidase-conjugated anti-His monoclonal antibody (Roche).

### One-step growth curves

One-step growth curve and burst-size determination were previously described [24]. The one step assays were carried out in *M. smegmatis* exponential growth cells using an MOI of 1. Cells were pelleted and resuspended in 1 ml of a phage suspension (Ms6*_wt_*, Ms6-Lysin_241_His_6_ or Ms6-Lysin_384_His_6_) supplemented with 1 mM CaCl_2_. The mixture was incubated 50 min at 37°C to allow adsorption of the phages. 100 µl of 0.4% H_2_SO_4_ was added to inactivate the non-adsorbed phages and the incubation continued for five min. The suspension was neutralized with 100 µl of 0.4% NaOH and diluted 1∶100 in 7H9 supplemented with 0.5% glucose and 1 mM CaCl_2_. 1 ml samples were withdrawn every 30 min until reaching 300 min. 100 µl of serial dilutions of each sample were plated with 200 µl of *M. smegmatis* cells, on 7H10 as top agar lawns and the phage titer for each sample was determined after 24 h incubation at 37°C. Results are averages of three independent experiments.

### Expression of Lysin_384_ and Lysin_241_ proteins in *E. coli*

*E. coli* BL21 (DE3):pMJC41 or *E. coli* BL21 (DE3):pMJC42 were grown in LB medium to an OD_600 nm_ of 0.6, and expression of the recombinant Lysin_384_-His_6_ or Lysin_241_-His_6_ was induced for 4 h following the addition of isopropyl β-D-1-thiogalactopyranoside to a final concentration of 1 mM. Bacterial cells were harvested by centrifugation, washed, resuspended in 50 mM Tris-HCl (pH 7.5) supplemented with a cocktail of protease inhibitors (Calbiochem), and disrupted by passage through a French pressure cell. Cell debris were removed by centrifugation, and the recombinant proteins present in the supernatant were analysed by SDS-PAGE, followed by Coomassie blue staining and Western blotting and detected as described above.

### Assay of the antibacterial activity of Lysin_384_ and Lysin_241_

The antibacterial activity was screened using a sensitivity test [28], [29] with some modifications. 100 µl of an exponential growing culture of the test strain was plated on LB or 7H10+OADC (for mycobacteria) as top agar lawns. 20 µl of induced *E. coli*:pMJC41 or *E. coli*:pMJC42 extracts containing Lysin_384_ or Lysin_241_ were spotted onto the bacterial lawn of the test strain and incubated overnight at 37°C. After overnight incubation, the presence of a clear zone was examined. *E. coli*:pET29b induced extract was used as a negative control. Activity assays were performed in triplicate. Several bacterial strains were used to test the range of antibacterial activity and were obtained from the American Type Culture Collection (ATCC) or from the Institute Pasteur Collection, Paris.

### Zymogram analysis

Sodium dodecyl sulfate (SDS)-polyacrylamide gel electrophoresis was performed as described by Laemmli (1970) [30], and the zymogram assays were carried out as outlined by Piuri and Hatfull (2006) [31]. Briefly, 0.2% autoclaved and lyophilized *Micrococcus luteus* cells were included in 15% polyacrylamide gels for detection of bacteriolytic activity. Protein samples were heated for 3 min at 100°C in sample buffer (62.5 mM Tris-HCl, pH 6.8; 2% SDS; 5% mercaptoethanol; 20% glycerol; 0.01% bromophenol blue), and then separated on SDS-gels containing the autoclaved cells. After electrophoresis, the zymograms were washed for 30 min with distilled water at room temperature and then transferred into renaturation buffer containing 25 mM Tris-HCl (pH 7.5) and 0.1% Triton X-100 followed by further incubation for 16 h at 37°C. The zymograms were rinsed with distilled water, stained with 0.1% methylene blue in 0.01% KOH for 2 h at room temperature, and then destained with distilled water. Peptidoglycan hydrolase activity was detected as a clear zone on a dark blue background of stained peptidoglycan. Gels not containing peptidoglycan were stained with Coomassie Brilliant Blue. Lysozyme and bovine serum albumin (BSA) were used as positive and negative controls, respectively. Molecular masses were determined by comparison with prestained molecular weight standards that were electrophoresed on the same gel.

## Results

### Identification of two gene products from Ms6 *lysA*

**Figure 1:**
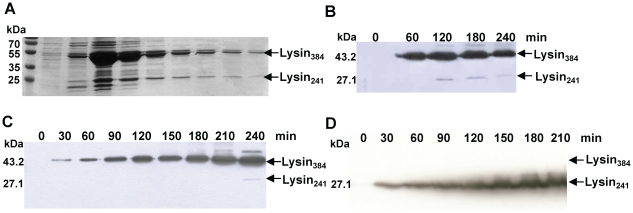
LysA expression in *E. coli*. **A.** Purified LysA-His_6_ fractions after SDS-PAGE analysis and Coomassie Blue staining. LysA-His_6_ was produced from pMJC41 in *E. coli* BL21 (DE3) after isopropyl β-D-1-thiogalactopyranoside induction. **B.** Expression of LysA-His_6_ from pMJC41: detection of C-terminal His_6_tag LysA shows the production of Lysin_384_-His_6_ and Lysin_241_-His_6_. **C.** LysA-His_6_ synthesis from pMJC41 over the time is not always followed by Lysin_241_-His_6_ production. **D.** Synthesis of Lysin_241_-His_6_ from pMJC43. Removal of pET29b and LysA (Lysin_384_-His_6_) translational signals does not hinder Lysin_241_-His_6_ synthesis. The molecular masses in kDa of Lysin_384_ and Lysin_241_ are indicated on the left; positions of both proteins are indicated by an arrow on the right. Lysin_384_ and Lysin_241_ were detected by Western blotting with an anti-His_6_ antibody, except for panel A.

The 1155 bp *lysA* gene of mycobacteriophage Ms6 starts at a GTG codon that overlaps the *gp1* TGA stop codon in a different reading frame, and is preceded in four nucleotides by a RBS sequence (5′-GGGAGCA-3′) (Fig. S1). It encodes a 384 amino acid polypeptide with significant similarity to bacteriophage encoded *N*-acetyl-muramoyl-L-alanine amidases [18]. During an attempt to purify LysA as a C-terminal histidine-tagged fusion product (LysA-His_6_), we detected the production of two proteins of ∼27 kDa (Lysin_241_) and ∼43 kDa (Lysin_384_), rather than a single polypeptide of 43 kDa, in *E. coli* crude extracts (Fig. 1A). We also observed that when the protein was tagged in the N-terminal domain, only the larger product (Lysin_384_) reacted with the anti-His_6_ antibody (data not shown). The fact that a time-dependent decrease of the 43 kDa form with a concomitant increase of the labelled 27 kDa form was not observed, rather both proteins seemed to be produced independently over the time (Fig. 1B), led us to consider that the smaller protein was not an N-terminal processed form of a larger precursor, but rather two independent translated products. Also supporting this notion, is the fact that analysis of the amino acid sequence of Ms6 LysA did not predict an amino-terminal signal sequence or a peptidase cleavage site [24]. Further examination of the *lysA* nucleotide sequence revealed a second potential gene entirely embedded in the same reading frame, preceded by a putative Shine-Dalgarno sequence (5′-TGGAGGT-3′) utilized by Gram-positive bacteria and mycobacteria [32], 6 nucleotides upstream of the GTG start codon (Fig. S1), and designated *lysA_241_*. Occurrence of an additional translation event at the predicted location would be compatible with the observed molecular masses (∼43 and 27 kDa) of the two proteins, considering that an additional His_6_tag was C-terminally fused to both proteins. This was experimentally confirmed by sequencing the amino-terminal region of the 27 kDa protein (Lysin_241_). This protein was obtained by expressing LysA in *E. coli*, followed by isolation of the smaller lysin from a polyvinylidene difluoride membrane. The obtained N-terminal sequence, MPDEPRPD, matched the deduced sequence from residues 144 to 151. Of note is the fact that, following expression in *E. coli*, the larger product (Lysin_384_) is in great excess when compared to the smaller protein (Lysin_241_) and frequently Lysin_241_ synthesis was not observed (Fig. 1C). To further clarify these results, *lysA* lacking its own GTG start codon was cloned into the XbaI/HindIII sites of pET29 which removes the Shine-Dalgarno sequence and the start codons of the expression vector. We were expecting that if Lysin_241_ results in fact from a new translation event and is not the result of cleavage of Lysin_384_, it should be synthesized from this construction. Western blotting analysis revealed the production of a single polypeptide with 27 kDa corresponding to Lysin_241_ (Fig. 1D). This result unambiguously proves that Lysin_241_ results from a new translation event and is independent of Lysin_384_ synthesis.

**Figure 2:**
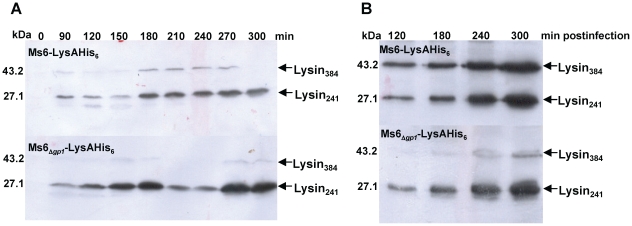
Time course of Lysin_384_ and Lysin_241_ synthesis during Ms6 infection of *M. smegmatis*. Lysin production in *M. smegmatis* was analysed after infection with Ms6-LysAHis_6_ or Ms6_Δ*gp1*_-LysAHis_6_ at an MOI of 10. Extracts were prepared from samples taken at 30-min intervals as described in Material and Methods. Samples were analysed by Western blotting and Lysin_384_ and Lysin_241_ synthesis was detected with an anti-His_6_ monoclonal antibody. **A.** Lysin_384_ and Lysin_241_ synthesis is first detected 90 minutes postinfection both in Ms6-LysAHis_6_ (upper panel) and Ms6_Δ*gp1*_-LysAHis_6_ (lower panel) mutant phages. Only the results for 90 to 300 min postinfection are shown. **B.** Lysin_384_ is synthesized to near undetectable levels during Ms6_Δ*gp1*_-LysAHis_6_ infection of *M. smegmatis* (lower panel) whereas Lysin_241_ production is comparable to the wild-type phage. The molecular masses in kDa of Lysin_384_ and Lysin_241_ are indicated on the left; positions of both proteins are indicated by an arrow on the right.

To follow mycobacteriophage Ms6 LysA production in the course of *M. smegmatis* infection, infected cells were examined for lysin synthesis. *M. smegmatis* was infected with a Ms6 derivative mutant, Ms6-LysAHis_6_ phage, where the 3′ end of *lysA* gene was fused to a sequence coding for a hexahistidine tag, allowing the production of a LysA-His_6_ tagged protein [24]. Samples were collected immediately before and every 30 minutes following infection until near the end of the Ms6 infection cycle. Protein extracts were prepared from such samples as described in materials and methods and checked for the presence of Histagged proteins by immunoblotting. Two proteins rather than a single lysin band, with 27 kDa and 43 kDa were detected, corresponding to the predicted molecular masses of Lysin_241_-His_6_ and Lysin_384_-His_6_, respectively (Fig. 2A). Both proteins were first detected at 90 minutes postinfection with mobilities indistinguishable from that exhibited by the protein forms of the *E. coli* expressed lysin. Taking into consideration that Lysin_384_ interacts with Ms6 Gp1, we followed the production of both proteins in an infection assay with an Ms6 mutant lacking *gp1* [24]. In this assay, we observed a decrease in Lysin_384_ although Lysin_241_ amount was comparable to the levels detected for the wild-type phage (Fig. 2B). Similarly to what was observed during Ms6 infection, both endolysins were detected 90 minutes postinfection (Fig. 2A).

These results suggest that during *M. smegmatis* Ms6 infection, different products of the *lysA* gene are synthesized and result from the existence of two translational events that direct the production of a smaller (Lysin_241_) and a larger (Lysin_384_) endolysin rather than a processing event. In addition, synthesis and/or stability of Lysin_384_ seem to be dependent on the Gp1 chaperone-like protein as already proposed [24].

### Occurrence of Ms6 LysA-like proteins in mycobacteriophages

All mycobacteriophage genomes sequenced so far possess putative *lysA*-like genes that were grouped in the mycobacteriophage gene family Pham66-1, although this group of enzymes is not restricted to phages that infect mycobacteria [33]. The Lysin A (LysA) family of proteins appears to be a particularly highly diverse and interesting group of lytic enzymes composed of subgenic modules with reasonably defined boundaries [34] containing different peptidoglycan hydrolase motifs including amidases, glycosidases and peptidases, as well as peptidoglycan-binding motifs [35]. Phages Ms6 and TM4 encode enzymes with an amid-2 type domain, while others such as D29 and Bxb1 employ hydrolases with lysozyme-like domains to bring about host cell lysis [23], [33]. A search for conserved domains showed that Ms6 LysA holds a central peptidoglycan recognition protein (PGRP) conserved domain (cd06583), localized between amino acid residues 168 and 312 (Fig. S2). PGRPs are pattern recognition receptors that bind, and in certain cases, hydrolyze peptidoglycan of bacterial cell walls. This family includes Zn-dependent *N*-acetylmuramoyl-L-alanine amidases (EC: 3.5.1.28) which cleave the amide bond between *N*-acetylmuramoyl and L-amino acids, preferentially D-lactyl-L-Ala, in bacterial cell walls.

The mycobacteriophage Ms6 lysis module is closely related to the lysis module of phages belonging to cluster F, subcluster F1, which includes phages PMC, Llij, Che8, Boomer, Fruitloop, Pacc40, Ramsey and Tweety [33], Ardmore [36] and Wee (GenBank accession number YP004123853). A BLASTp search for Ms6 LysA homologues identified similar proteins amongst phages of this subcluster, with a high level of identity, except for the endolysin of mycobacteriophage Pacc40, and produced significant alignments with the N-terminal region of endolysins belonging to unclustered mycobacteriophages Corndog, Phyler, Phaedrus and Pipefish and with the endolysin of phage LeBron (GenBank accession number YP003857156) [37]. Examination of the endolysin nucleotide sequences of these bacteriophages revealed the existence in all of them of a second potential translation site positioned in close proximity to the beginning of the central PGRP domain (Fig. S2, Table 2). As recently described, the accessory lysis protein Gp1 binds the N-terminal domain of LysA [24] and *gp1* was implicated in lysis because of its linkage to *lysA* and the demonstration of its requirement for mycobacteria efficient lysis [24]. *gp1*-like genes are present in many, but not all mycobacteriophage genomes and were grouped in the mycobacteriophage gene family Pham1480 [33]. In addition to subcluster F1, homologues of Ms6 Gp1 were also identified in all phages belonging to subcluster A1. However phages Lockley and Jasper did not reveal the existence of two potential translational sites in the *lysA* gene. Members of Pham1480 are mostly found adjacent to the Pham66-1 encoding genes, suggesting an even more intimate association between the gene products: while in some phages of subcluster A1 and of subcluster F1, *gp1*-like genes are overlapped with the lysin gene, in phages DD5, KBG and Solon (subcluster A1) and in phages Boomer and Ramsey (subcluster F1), Pham1480 is separated from the lysin gene by one intervening gene that code for putative homing endonuclease HNH motifs [33]. Although in these mycobacteriophages *gp1* and *lysA* are closely linked, several other mycobacteriophages seem to have somewhere in their genomes genes encoding proteins that could perform functions analogous to Gp1, while others simply lack *gp1* [33].

**Table 2:** Occurrence of Ms6 *lysA*-like genes in mycobacteriophages.

Mycobacteriophage	*E. coli* consensus			Start CodonPosition*
	SD		Start Codon	
	AGGAGGT	4-7	→ATG	
Cluster A/subcluster A1				
Bxb1	CAAGGA	tgcg	ATG	397 (1434 bp)
U2	GAAGGA	cgcg	ATG	397 (1434 bp)
Bethlehem	GAAGGA	cgcg	ATG	397 (1434 bp)
Solon	AAAAGA	tgcg	ATG	379 (1395 bp)
KBG	AAAAGA	tgcg	ATG	379 (1395 bp)
DD5	AAAAGA	tgcg	ATG	379 (1395 bp)
SkiPole	AAAAGA	ccca	ATG	397 (1413 bp)
ClusterB/subcluster B3				
Pipefish	AGGAGG	acagctccc	GTG	478 (1224 bp)
Phaedrus	AGGAGG	acagcgccc	GTG	322 (1068 bp)
Phyler	AGGAGGC	acagcgccc	GTG	520 (1266 bp)
Cluster F/subcluster F1				
Ms6	TGGAGGT	accgcc	GTG	430 (1155 bp)
Che8	CGGAGGA	acct	GTG	514 (1275 bp)
Tweety	TGGAGGT	accgcc	GTG	430 (1212 bp)
PMC	TGGAGGT	accgcc	GTG	430 (1194 bp)
Llij	TGGAGGT	accgcc	GTG	430 (1194 bp)
Boomer	CGGAGGT	tccc	ATG	523 (1302 bp)
Fruitloop	TGGAGGT	accgcc	GTG	430 (1155 bp)
Ramsey	CGGAGGA	acct	GTG	514 (1248 bp)
Pacc40	AGGAGAG	gacgcaaac	GTG	442 (1209 bp)
Wee	TGGAGGT	accgcc	GTG	430 (1155 bp)
Ardmore	TGGAGGT	accgcc	GTG	430 (1155 bp)
Unclustered				
LeBron	TGAGGT	aatc	GTG	448 (1173 bp)
Corndog	GGGAGGA	aca	GTG	439 (1221 bp)

*Numbers refer to nucleotide positions. The gene size is indicated in parenthesis.

### Role of Lysin_384_ and Lysin_241_ during *M. smegmatis* phage infection

For a better understanding of the contribution of the endolysin to the Ms6 infection cycle, we first investigated whether its hydrolase activity is an essential function for host lysis. We used the Bacteriophage Recombineering of Electroporated DNA (BRED) strategy [27] to delete *lysA* gene (*gp*2) from the mycobacteriophage Ms6 genome. Oligonucleotides were designed to introduce a 1089 bp internal-deletion in Ms6 *lysA*, fusing 19 codons at the 5′ and 3′ends of the gene to maintain the *lysB* RBS and minimize effects on expression of the adjacent *lys*B gene, as well as avoiding genetic polarity. As expected, *lysA* was essential for mycobacterial lysis and no viable phages could be recovered from a deletion of Ms6 *lysA* gene (data not shown). The same result was also reported for mycobacteriophage Giles [27].

**Figure 3:**
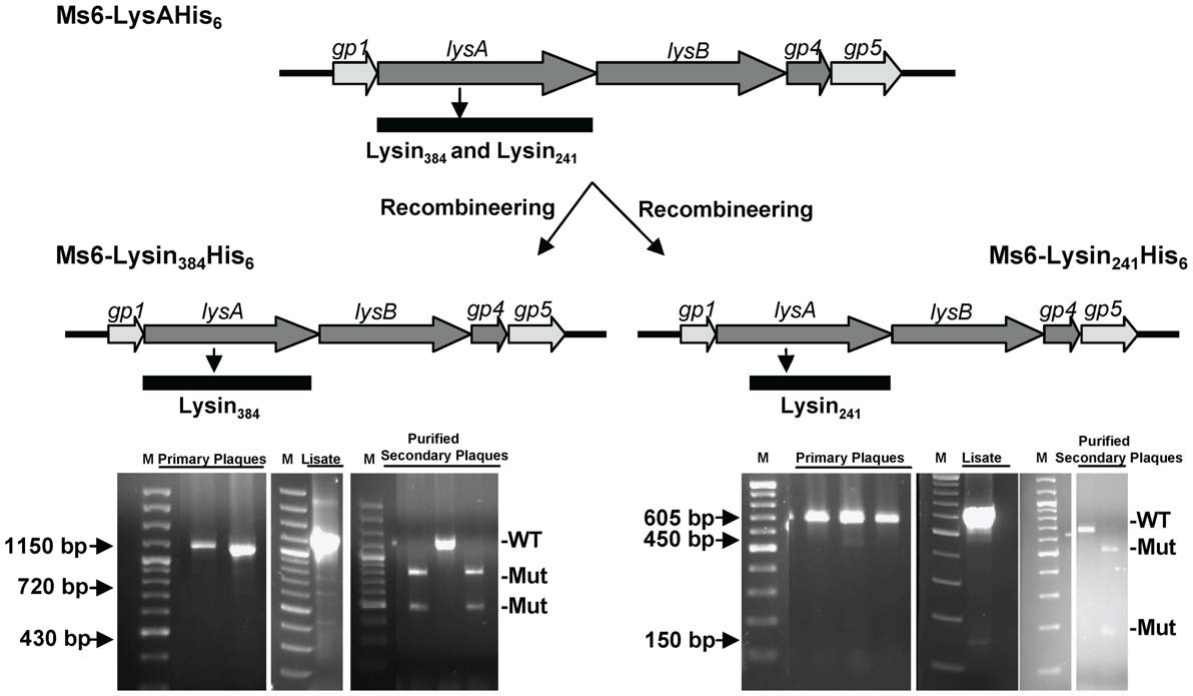
Construction of Ms6 *lysA* mutants. Two complementary oligonucleotides that modify *lysA*_241_ GTG start codon (valine) to TGG (tryptophan) and introduce an MscI restriction site, or two complementary oligonucleotides that introduce a stop codon and a HindIII restriction site downstream of the start codon of *lysA*_384_, were co-transformed with Ms6-LysAHis_6_ genomic DNA; primary plaques were recovered and screened by PCR and MscI or HindIII digestion to identify a mixed plaque containing wild-type and mutant phages DNA. The mixed primary plaque was diluted and plated; the lysate was screened to check for phage viability, and purified secondary plaques were screened to identify pure mutant phages of Ms6-Lysin_384_His_6_ and Ms6-Lysin_241_His_6_, expressing only Lysin_384_ or Lysin_241_, respectively.

**Figure 4:**
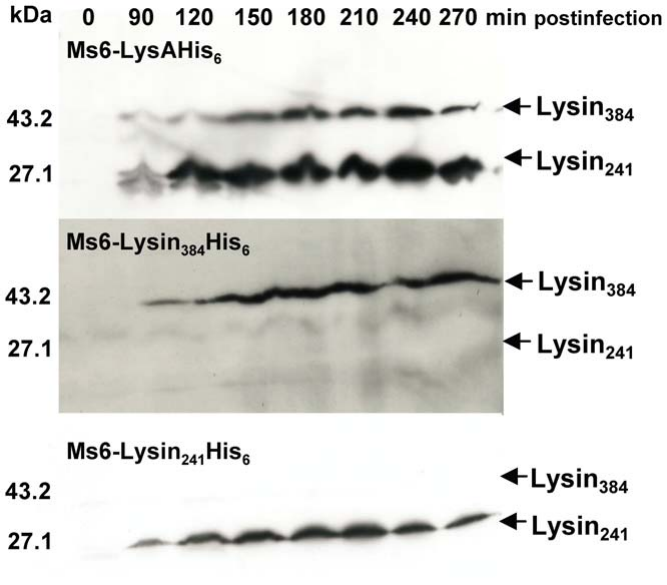
Time course of endolysin synthesis during Ms6-LysAHis_6_, Ms6-Lysin_384_His_6_ or Ms6-Lysin_241_His_6_ infection of *M. smegmatis*. Lysin production in *M. smegmatis* was analysed after infection at an MOI of 10. Extracts were prepared from samples taken at 30-min intervals as described in Material and Methods. Samples were analysed by Western blotting and Lysin_384_ and Lysin_241_ synthesis was detected as already described. Both Lysin_384_ and Lysin_241_ synthesis could be detected beginning 90 minutes postinfection in Ms6-LysAHis_6_ (upper panel). In Ms6-Lysin_384_His_6_ and Ms6-Lysin_241_His_6_, (lower panels) only Lysin_384_ or Lysin_241_ synthesis could be detected also beginning 90 min postinfection, respectively. Only the results for 90 to 270 min postinfection are shown. The molecular masses in kDa of Lysin_384_ and Lysin_241_ are indicated on the left; positions of both proteins are indicated by an arrow on the right.

**Figure 5:**
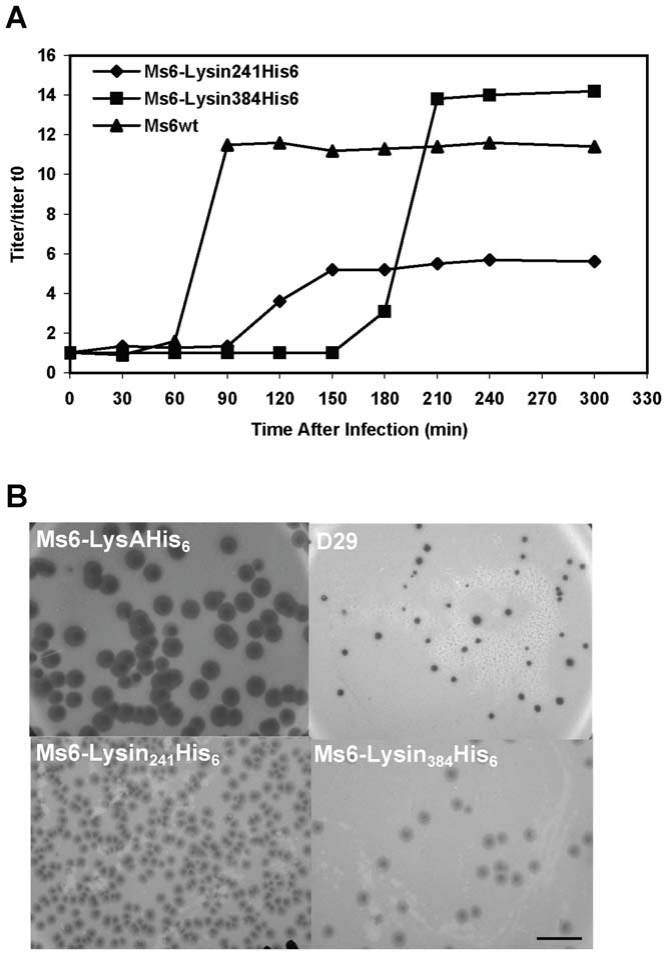
Both Lysin_384_ and Lysin_241_ are required for efficient host cell lysis. **A.** One step growth curves of Ms6 and lysin-mutant derivatives. For each curve the titers measured were divided by the titer at t = 0 for normalization (titer/titer t_0_). **B.** Halo formation by mycobacteriophage Ms6 and lysin-mutant derivatives. Serial dilutions of the bacteriophages stocks containing ∼10^10^ particles ml^−1^ were prepared and 100 µl of the 10^−8^ dilution was plated with 200 µl of an exponential growing culture of *M. smegmatis* as top agar lawns. Incubation was continued for 3 days at 37°C. Mycobacteriophage D29 was used as a negative control of halo formation [36]. Scale bar represents 1 cm.

As a result of synthesis of Lysin_241_ and Lysin_384_ during phage infection, we next investigated the influence of each endolysin form in phage growth parameters. Once more, we took advantage on the fact that the BRED recombineering strategy has already been described to efficiently introduce base changes that confer an amino acid substitution [27]. To eliminate synthesis of Lysin_384_ or Lysin_241_ we designed oligonucleotides that introduce a stop codon and a HindIII restriction site downstream of the start codon of *lysA_384_*, or two complementary oligonucleotides that modify the *lysA_241_* GTG start codon to TGG (tryptophan), and introduce an MscI restriction site, respectively. Both mutant phages Ms6-Lysin_384_His_6_ (producing Lysin_384_) and Ms6-Lysin_241_His_6_ (producing Lysin_241_) were readily isolated, demonstrating that for plaque formation only one of the two LysA forms, Lysin_384_ or Lysin_241_ is required (Fig. 3). Nevertheless, we considered whether the absence of one of the two endolysin forms during *M. smegmatis* phage infection (Fig. 4) could confer an altered lysis phenotype. To address this question one step growth curves and determination of phage growth parameters (latent period, rise period and burst size) were carried out to compare the phages infection cycle. The one step growth experiment (Fig. 5A) shows that in a phage Ms6-Lysin_241_His_6_ infection, the latent period is prolonged 30 min in comparison with the Ms6*_wt_* infection and a decrease in the burst size was observed which means that a delay exists in the detection of phage release from cells infected with Ms6-Lysin_241_His_6_. In agreement, absence of Lysin_384_ leads to smaller size phage plaques (Fig. 5B), meaning that Lysin_384_ is important for infective particles release. On the other hand, Ms6-Lysin_384_His_6_ phage release starts 90 min later than with Ms6*_wt_*. This indicates that similarly to Lysin_384_, Lysin_241_ has an obvious function in completion of lysis, although it does not have a significant effect in the number of phage particles released. Examination of Ms6-Lysin_384_His_6_ phage plaques shows that although not larger in size, plaques are more turbid than wild-type Ms6 probably due to a partial host cell lysis (Fig. 5B). These results strongly suggest that even though only one of the two LysA forms, Lysin_384_ or Lysin_241_, is required to accomplish host cell lysis, both enzymes are necessary for complete and efficient lysis of *M. smegmatis*.

### Lysin_384_ and Lysin_241_ are cell wall-degrading enzymes with peptidoglycan hydrolase activity

**Figure 6:**
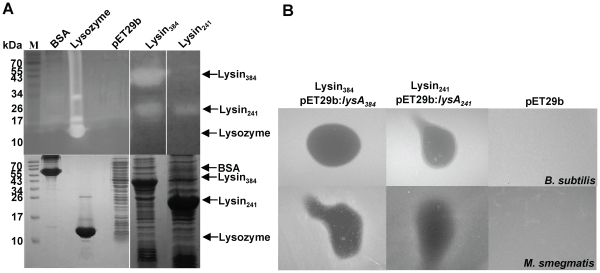
Peptidoglycan hydrolysis by *E. coli*-produced Lysin_384_ and Lysin_241_ in *M. luteus* cells. **A.** Lytic activity of lysin extracts was assessed by *in situ* renaturation after SDS-PAGE using a gel matrix containing *M. luteus* cells as substrate (upper panel). Peptidoglycan hydrolysis by renatured proteins within the gel produces clear zones that no longer stain with methylene blue. Lysozyme and bovine serum albumin (BSA) represent positive and negative controls, respectively. A cell-free control gel was run in parallel and stained with Coomassie blue (lower panel). The molecular masses in kDa of BSA, lysozyme, Lysin_384_ and Lysin_241_ are indicated on the left; positions of proteins are indicated by an arrow on the right. **B.** Effect of Lysin_384_ or Lysin_241_ activity on lawns of *B. subtilis* (upper panel) and *M. smegmatis* (lower panel). 20 µl of *E. coli*:pMJC41 or *E. coli*:pMJC42 extracts containing Lysin_384_ or Lysin_241_ were spotted onto the bacterial lawn of the test strain and incubated overnight at 37°C. After overnight incubation, the presence of a clear zone was examined. *E. coli*:pET29b induced extract was used as a negative control.

LysA has been previously described as not affecting *E. coli* growth rate unless permeabilization of the plasma membrane by chloroform addition which results in immediate lysis [18]. To follow growth and viability of *E. coli* strains expressing Lysin_241_, the *lysA* DNA fragment corresponding to *lysA*_241_ was cloned into pQE30 vector allowing expression of Lysin_241_ under the control of the regulated T5 bacteriophage promoter. Although induction of Lysin_241_ did not result in *E. coli* lysis unless chloroform was added, growth seems to be halted over the induction period (data not shown). Ms6 LysA holds a PGRP domain and its hydrolase activity was already demonstrated; the protein was shown to cleave the bond between L-Ala and D-muramic acid and to release up to 70% of the diaminopimelic acid present in isolated mycobacterial cell walls which confirmed the amidase activity of the enzyme (unpublished results). To more directly assess the enzymatic activity of Lysin_384_ and Lysin_241_ we tested their ability to generate a zone of clearing in a zymogram assay. Lytic activity in lysin-producing *E. coli* extracts was checked by *in situ* protein renaturation after SDS-PAGE, using gel-incorporated autoclaved *M. luteus* cells as the substrate. As shown in Fig. 6A both Lysin_384_ and Lysin_241_ have hydrolase activity. Peptidoglycan hydrolysitic activity in zymograms was already demonstrated for other three mycobacteriophage LysA proteins (Che8 Gp32, Bxz1 Gp236 and Corndog Gp69) [22] and TM4 Gp29 [38].

When Ms6 was first isolated, it was noticed that it forms plaques in *M. smegmatis* lawns with halos surrounding them. Formation of the halo begins after two days of incubation, once the infected area is fully formed. Halo formation around phage plaques seems to be unusual among mycobacteriophages. Nevertheless, this phenomenon has been described for, at least, mycobacteriophages Bxb1 [39] and Che12 [40] and has been observed for a number of other phages, particularly those infecting Gram-positive bacteria; plaques of phage A511 infecting *Listeria monocytogenes* were shown to form clearly visible secondary lysis zones caused by release of Ply511, a hydrophilic amidase endolysin that diffuses from the centre of the plaque and hydrolyses the surrounding cells “from without” [7]. *M. smegmatis* cells in the exponential growth phase were infected with phages D29, Ms6*_wt_*, Ms6-Lysin_241_His_6_ or Ms6-Lysin_384_His_6_. Halo formation around D29 phage plaques was not observed (Fig. 5B) while Ms6*_wt_* forms turbid plaques showing size variation with partially clear halos surrounding them (Fig. 5B). When we analysed halo formation in *M. smegmatis* infected with phages Ms6-Lysin_241_His_6_ or Ms6-Lysin_384_His_6_, we observed that both phages retain the ability to form halos, although smaller and much more turbid than the wild-type phage halo (Fig. 5B). It is possible that these halos might result from the diffusion of Lysin_384_ or Lysin_241_ from the Ms6*_wt_* phage infective centre, leading to a “lysis from without” in surrounding cells. As both endolysins forms of LysA are essential to obtain an effective lysis of *M. smegmatis*, halo turbidity could result from partial lysis of cells surrounding the phage plaques. However, these results do not rule out the existence in the Ms6 genome of other genes whose products could be implicated in the halo formation phenomenon.

Despite the fact that Ms6 LysA is produced by a phage that infects a bacterium with a complex envelope and taking into consideration the above results, we tested the activity of *E. coli* crude extracts containing Lysin_384_ or Lysin_241_ in mycobacteria and other bacterial species by spotting the extracts on lawns of exponential growing cells. The results, summarized in Table 3, showed that *E. coli* extracts containing Lysin_384_ or Lysin_241_ inhibited the growth of all mycobacteria tested except *M. fortuitum*, as well as the Gram-positive bacteria: *M. luteus*, *M. pyogenes*, *B. subtilis* and *B. pumilus*. A weak activity was observed against *S. pyogenes*, *S. aureus*, *S. epidermidis* and the Gram-negative bacterium *E. coli*. For each strain tested, a crude extract of *E. coli* BL21 (DE3):pET29b was used as a control, and no inhibition was observed for any bacterial strain (Fig. 6B). Activity on mycobacteria cells was also confirmed by the ability of bacteriophages Ms6*_wt_*, Ms6-Lysin_241_His_6_ and Ms6-Lysin_384_His_6_ to form clearing zones on lawns of growing cells with *M. smegmatis* and *M. aurum* showing to be the most sensitive mycobacteria (Table 3).

**Table 3:** Antibacterial activity of Ms6 and derivative mutants and its lysis proteins.

	Bacteriophage activity*			Protein activity*	
	Ms6_wt_	Ms6-Lysin_241_His_6_	Ms6-Lysin_384_His_6_	Lysin_384_	Lysin_241_
*Mycobacterium smegmatis*	++	++	++	++	++
*Mycobacterium vaccae*	+	+	−	++	++
*Mycobacterium aurum*	++	++	−	++	++
*Mycobacterium fortuitum*	+	+	−	−	−
*Enterococcus faecium*	−	−	−	−	−
*Enterococcus faecalis*	−	−	−	−	−
*Streptococcus pyogenes*	−	−	−	+	+
*Micrococcus luteus*	−	−	−	++	++
*Micrococcus pyogenes*	−	−	−	++	++
*Bacillus subtillis*	−	−	−	++	++
*Bacillus pumilus*	−	−	−	++	++
*Staphylococcus aureus*	−	−	−	+	+
*Staphylococcus epidermidis*	−	−	−	+	+
*Escherichia coli*	−	−	−	+	+

*Designations refer to bacterial lawn clearance and are as follows: ++, clearance observable at the site of bacteriophage or lytic protein application; +, partial clearance observable at the site of bacteriophage or lytic protein application; −, no clearance.

## Discussion

In the present work, we provide evidence that two proteins (Lysin_384_ and Lysin_241_) with endolysin activity are produced from the mycobacteriophage Ms6 *lysA* gene. Our group has recently described that the N-terminal domain of Lysin_384_ is necessary and sufficient to directly interact with Gp1, a chaperone-like protein located upstream of LysA that assists the translocation of the endolysin across the cytoplasmic membrane in a holin-independent way [24]. Evidence for the presence of two forms of LysA raised the possibility that the shorter protein could be the mature form of Lysin_384_: if a cleavage event had occurred, it would implicate the removal of the N-terminal 143 amino acids of the enzyme corresponding to 16 kDa. Although unusual, as generally mycobacterial SP length is 32 residues [41], similar to the lengths of SPs from Gram-positive bacteria [42], cleavage of a large segment of 143 amino acids that functions as a SP has already been described for the *Staphylococcus simulans* lysostaphin [43]. However, no sequence signals or cleavage sites were detected by bioinformatic analysis in the N-terminal region of LysA [24]. Examination of the *lysA* nucleotide sequence revealed the existence of a second possible lysin gene embedded within *lysA* in the same reading frame and preceded by a consensus Shine-Dalgarno sequence (Fig. S1). The existence of two translation events was clearly demonstrated when Lysin_241_ was still produced after elimination of the translation signals from Lysin_384_, both in *E. coli* and in *M. smegmatis* phage infection: if Lysin_241_ was a processed form of Lysin_384_ it could not be detected. Thus, occurrence of a cleavage event, as observed for the endolysin of bacteriophage fOg44 [13] was ruled out. Coding sequences entirely encompassed within other genes seem to be very rare among dsDNA bacteriophages: λ*rz* and *rz1* represent an example of two genes located in different reading frames in the same nucleotide sequence which encode different proteins, both required in the same physiological pathway [44]. Although very rare, some exceptions among bacteriophage endolysins were found: 1) the bacteriophage φvML3 endolysin gene encodes two proteins, a larger lysin that has homology with lysozymes and a smaller lysis protein that has some features resembling those of a holin [45]; 2) the streptococcal C1 bacteriophage lysin called PlyC is a multimeric lysin composed of two separate gene products, PlyCA and PlyCB responsible for the hydrolytic amidase activity and cell-wall-binding domain, respectively [46]; 3) examination of the nucleotide sequence of bacteriophage CMP1 endolysin gene revealed a possible Shine-Dalgarno sequence within the gene, four nucleotides upstream of a second ATG codon in the same reading frame which would correspond to a gene product consisting of 166 C-terminal amino acid residues that includes the binding domain of the enzyme [47].

A BLASTn search for Ms6 *lysA* homologues revealed that this peculiar endolysin gene arrangement is widespread in mycobacteriophages, in particular amongst those that possess a *gp1*-like gene (Pham1480) [33], which would suggest that Gp1 may confer a selective advantage for host cell lysis under different environmental conditions: very small differences in lysis timing and efficiency are strongly selective because of competition for hosts by newly released progeny [48]. However, two putative translational signals were also identified in endolysin genes belonging to five mycobacteriophages (Phyler, Phaedrus, Pipefish, Corndog and LeBron) that do not possess Gp1 homologues but possess related Ms6 LysA sequences. The lack of representation of Pham1480 upstream of *lysA* in these phages could result from loss of *gp1*-like gene in these genomes. Furthermore, in three mycobacteriophages (TM4, Jasper and Lockley) that possess Gp1 similar proteins but unrelated Ms6 LysA enzyme, this *lysA* gene arrangement was not observed which suggests that Pham1480 in these mycobacteriophages might result from recent acquisition by horizontal genetic exchange [33]. These data also support the idea that all of these genomes have been in genetic communication, as Pham1480 is restricted to mycobacteriophages [33], and reflect the highly sequence diversity and modular nature of mycobacteriophage genomes that are characteristically mosaic comprising modules (frequently containing just a single gene) or cassettes, many of which shared by other phage genomes [49], [50]. Although we have clearly shown that Ms6 produces two endolysins and that both are required for lysis, a question remains to be answered: why mycobacteriophages need to produce two endolysins, or is this phenomenon only a consequence of gene transfer through evolution? More studies with other mycobacteriophages will certainly help to clarify the need for two endolysins and for Gp1 homologues.

Although a clear relationship between Gp1 and LysA does not seem to occur among all mycobacteriophages, our results indicate that in Ms6 a tight association between the two proteins exists. We observed that synthesis and/or stability of the larger endolysin (Lysin_384_) is highly dependent on Gp1 production. A reasonable explanation is that in the absence of its chaperone, the endolysin becomes unstable in the cytoplasm, or that an efficient translation of LysA is more or less dependent upon translation of the adjacent *gp1*-coding region as suggested by overlapping stop/start codons: Lysin_384_ expression may rely on the ribosome frameshifting at the GTGA sequence joining the *gp1* and *lysA* reading frames. When analysing LysA production during the infective cycle of both Ms6-LysAHis_6_ and Ms6_Δ*gp1*_-LysAHis_6_ phages, we observed a decrease in the Lysin_384_ levels in cells infected with Ms6_Δ*gp1*_, although Lysin_241_ synthesis remains apparently unaffected. However, this is not the result of a polar effect at the transcriptional level as infection of *M. smegmatis* cells expressing the wild-type Gp1 protein in trans, with Ms6_Δ*gp1*_, leads to a reversion of the lysis defect [24]. Construction of Ms6 mutant phages deleted in *lysA* or defective for Lysin_384_ or Lysin_241_ synthesis showed that LysA is essential for host cell lysis. In fact, as pointed out by R. Young (2005) [48] endolysins are always essential (for dsDNA phages) in terms of plaque-forming ability, whereas holins may be not; indeed, for mycobacteriophage Ms6, LysA is the only lysis function that can not be suppressed and is indispensable for lysis, even though deletion of the additional lysis genes (*gp1*, *lysB*, *gp4* and *gp5*) may result in poor phage viability and severe lysis defects [19], [24; Catalão and Gil, unpublished results]. Suppression of Lysin_384_ or Lysin_241_ synthesis does not result in a non-lysis phenotype as both proteins harbour the PGRP domain. However, lack of Lysin_384_ or Lysin_241_ in phage virion results in an altered lysis phenotype; analysis of the Ms6-Lysin_241_His_6_ (defective for Lysin_384_ synthesis) and Ms6-Lysin_384_His_6_ (defective for Lysin_241_ synthesis) phage growth parameters revealed that, whereas Lysin_384_ is necessary to achieve a normal burst of infective phages, Lysin_241_ has an important function in the progression and complete host cell lysis. At this time it is unknown if Lysin_384_ activation is dependent on holin function It is possible that Gp1 plays a role in maintenance of Lysin_384_ inactive state: Gp1 binding to the N-terminal domain may alter the endolysin conformation and block substrate binding or Gp1 may allow Lysin_384_ to adopt an active conformation. Indeed, the fact that Lysin_384_ is detected almost exclusively in the presence of Gp1 suggests that Gp1 might affect the stability of the endolysin. Chaperone-synthesis/stability dependence has been already described for some lipases [51]–[54]. The energized state of the cytoplasmic membrane was also described as being implicated both in autolysins activation [55]–[58] and secretory endolysins activation [13], [15], [59].

Remarkably, *E. coli* extracts containing Lysin_384_ or Lysin_241_ enzymes inhibited bacterial growth of most of the Gram-positive bacteria and mycobacteria tested (that included *M. smegmatis*, *M. vaccae*, *M. aurum* and *M. fortuitum*) contrary to what was previously thought [22], [36]. This data is in agreement with the ability of Ms6 to form turbid plaques surrounded by a clear zone of apparent bacterial growth inhibition. This phenomenon is widely observed among bacteriophages that infect Gram-positive hosts and results from “lysis from without” of bacteria as a result of endolysin diffusion from phage plaques that kills uninfected cells [7]. Although *Mycobacterium* spp. are Gram-positive bacteria included in the suborder of *Corynebacterineae*, the envelope of this bacterial group is composed of a typical plasma membrane surrounded by a cell wall core, which, in turn, is surrounded by an outer membrane layer [60], [61].

Unexpectedly, we observed that different mycobacterial species are susceptible to exogenously added Lysin_384_ and Lysin_241_, despite their mycolic-acid-rich outer membrane. Even though it is unlikely that Lysin_384_ or Lysin_241_ can diffuse through water-filled channels, the porins [62], [63], as typically only molecules with masses up to 600 Da can pass through the pores [64], it is possible that lysin access to the peptidoglycan may occur during cell division and septal peptidoglycan biogenesis. This is of interest as exogenously applied phage-encoded endolysins have been shown to possess effective antimicrobial activity [65] against Gram-positive bacterial pathogens [66]–[69]. The ultimate challenge will be engineer improved mycobacteriophage lysins with higher activity and test the synergistic effect with other enzymes as LysB or outer membrane permeabilizers that could facilitate the access of LysA to the peptidoglycan.

## Supporting Information

Figure S1 **Relevant features of the DNA sequence including and surrounding the Ms6** ***lysA*** **gene.** Putative RBS consensus sequences from *lysA*_384_ and *lysA*_241_ are shown in bold and underlined. Translational start and stop codons are superscripted and/or in bold. Amino acids residues of LysA are indicated below the nucleotide sequence. Amino acid substitutions and insertions to construct Ms6 *lysA* mutant phages are highlighted; Ms6-Lysin_241_His_6_ has a stop codon and a HindIII restriction site, downstream of the *lysA* start codon which eliminates synthesis of Lysin_384_; substitution of the GTG codon by TGG at position 144 eliminates synthesis of Lysin_241_ (Ms6-Lysin_384_His_6_); Ms6-LysAHis_6_ [23] and Ms6_Δ*gp1*_-LysAHis_6_ have a five histidine insertion just before the TGA stop codon to generate a His_6_tag C-terminal fusion with *lysA*.
(TIF)

Figure S2 **CLUSTALW alignment of Ms6 LysA and putative LysA amino acid sequences of subcluster F1 mycobacteriophages.** Mycobacteriophages: Llij Gp30 (YP655026), PMC Gp30 (YP655791), Ms6 Gp2 (AAG48318), Fruitloop Gp29 (YP002241714), Ardmore Gp29 (YP003495170), Tweety Gp30 (YP001469263), Wee Gp31 (YP004123853), Che8 Gp32 (NP817370), Boomer Gp32 (YP002014248) and Ramsey Gp32 (YP002241819); the primary accession numbers of the UniProtKB/TrEMBL database are given in parenthesis. Identical (*), highly similar (:) and similar (.) amino acids are indicated. Dashes represent gaps introduced by CLUSTALW to optimize the alignment. The PGRP conserved domain is highlighted on a grey background. Numbers refer to amino acid positions. Predicted start codons are shown in bold.
(TIFF)

Table S1 Oligonucleotides used in this study.
(DOC)
