# Supplementary figures and images for: A Second Endolysin Gene Is Fully Embedded In-Frame with the lysA Gene of Mycobacteriophage Ms6
Source: PLoS One. 2011 Jun 9;6(6):e20515. doi: 10.1371/journal.pone.0020515 (PMC3111421; doi:10.1371/journal.pone.0020515)

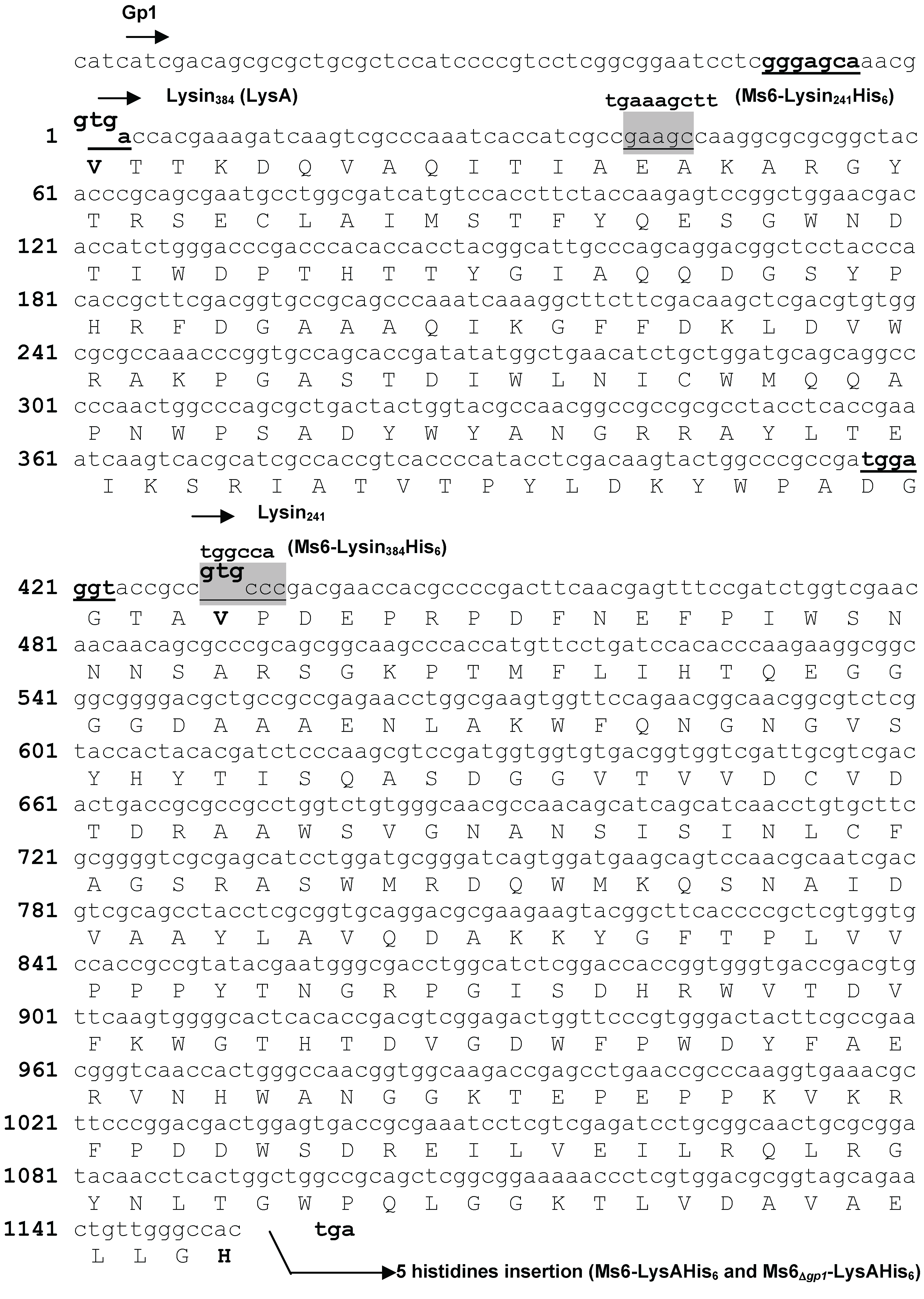

Supplement: Figure S1 — Relevant features of the DNA sequence including and surrounding the Ms6 lysA gene. Putative RBS consensus sequences from lysA 384 and lysA 241 are shown in bold and underlined. Translational start and stop codons are superscripted and/or in bold. Amino acids residues of LysA are indicated below the nucleotide sequence. Amino acid substitutions and insertions to construct Ms6 lysA mutant phages are highlighted; Ms6-Lysin241His6 has a stop codon and a HindIII restriction site, downstream of the lysA start codon which eliminates synthesis of Lysin384; substitution of the GTG codon by TGG at position 144 eliminates synthesis of Lysin241 (Ms6-Lysin384His6); Ms6-LysAHis6 [23] and Ms6Δgp1-LysAHis6 have a five histidine insertion just before the TGA stop codon to generate a His6tag C-terminal fusion with lysA. (TIF) [file pone.0020515.s001.tif]

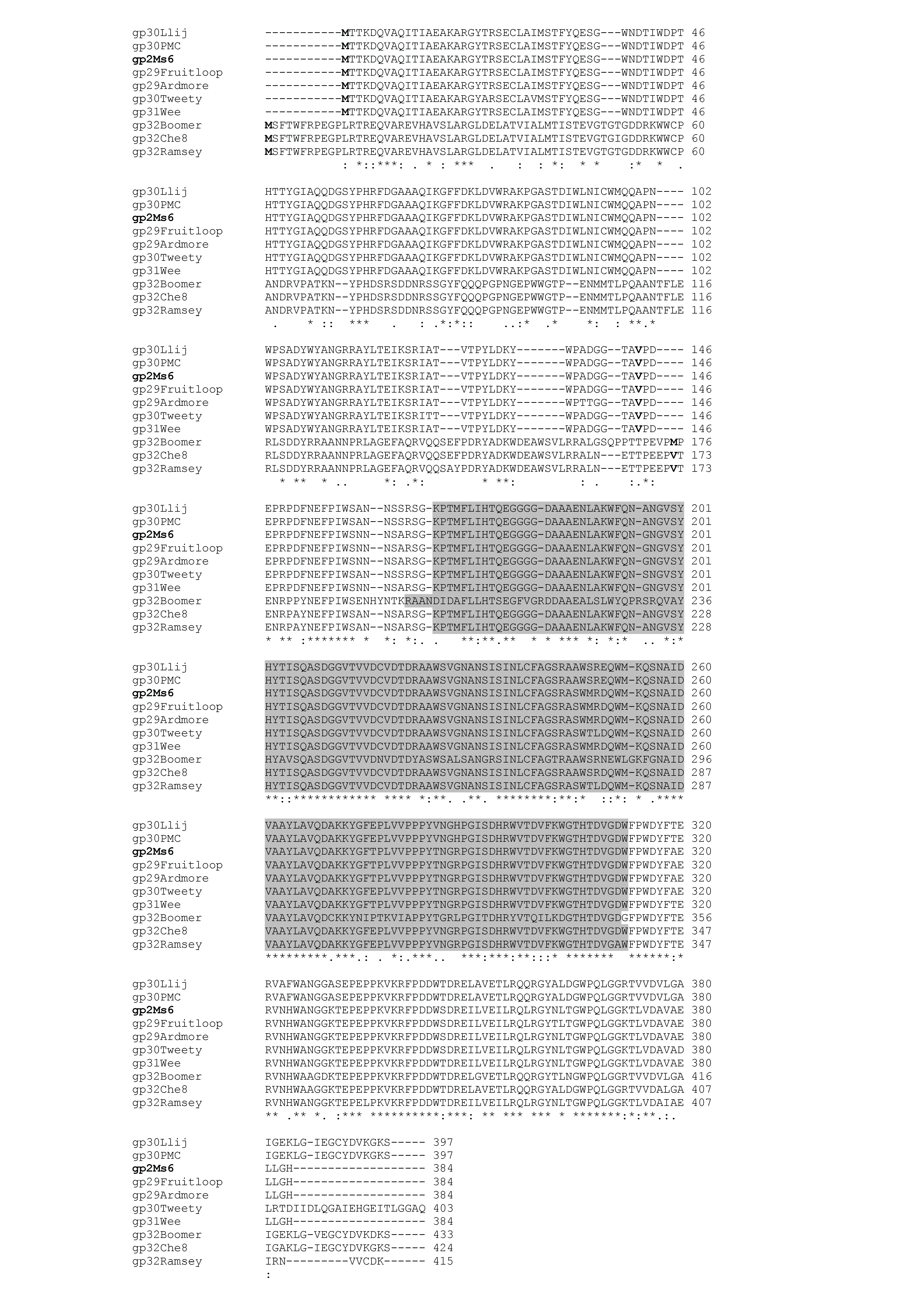

Supplement: Figure S2 — CLUSTALW alignment of Ms6 LysA and putative LysA amino acid sequences of subcluster F1 mycobacteriophages. Mycobacteriophages: Llij Gp30 (YP655026), PMC Gp30 (YP655791), Ms6 Gp2 (AAG48318), Fruitloop Gp29 (YP002241714), Ardmore Gp29 (YP003495170), Tweety Gp30 (YP001469263), Wee Gp31 (YP004123853), Che8 Gp32 (NP817370), Boomer Gp32 (YP002014248) and Ramsey Gp32 (YP002241819); the primary accession numbers of the UniProtKB/TrEMBL database are given in parenthesis. Identical (*), highly similar (:) and similar (.) amino acids are indicated. Dashes represent gaps introduced by CLUSTALW to optimize the alignment. The PGRP conserved domain is highlighted on a grey background. Numbers refer to amino acid positions. Predicted start codons are shown in bold. (TIFF) [file pone.0020515.s002.tiff]
