# Supplementary material for: A Second Endolysin Gene Is Fully Embedded In-Frame with the lysA Gene of Mycobacteriophage Ms6
Source: PLoS One. 2011 Jun 9;6(6):e20515. doi: 10.1371/journal.pone.0020515 (PMC3111421; doi:10.1371/journal.pone.0020515)
Supplement: Table S1 — Oligonucleotides used in this study. (DOC) [file pone.0020515.s003.doc]

**Table S1.** Oligonucleotides used in this study.

| **Oligonucleotide** | **Sequence^a^ 5´-3´** | **Use** |
| --- | --- | --- |
| *lysA_241_*fwd-1 | CAGGATCCCCCGACGAACCACGCCCC | Ms6 *lysA_241_*^CP^ pQE30 |
| *lysA_241_*fwd-2 | CAGGATCCCGACGAACCACGCCCC | Ms6 *lysA_241_*^CP^ pET29b |
| gp2A | CGCGGATCCCACCACGAAAGATCAAG | Ms6 *lysA_384_^C^*^P^ pET29b |
| pORF2-c1 | ATGCGAAGCTTCAGTGGCCCAACAGTTC | Ms6 *lysA_241_*^CP^ |
| p*lysA*ΔGTGfwd | GCTCTAGACCACGAAAGATCAAGTCGC | Ms6 *lysA_384_*_ΔGTG_^CP^  pET29b |
| Pr∆*lysA* | CTGCGCTCCATCCCCGTCCTCGGCGGAATCCTCGGGAGCAAACGGTGACTGGCCGCAGCTCGGCGGAAAAACCCTCGTGGACGCGGTAGCAGAACTGTT | Ms6 *lysA* 1089bpΔ |
| PrExtender∆*lysA*fwd | CGACCTGACCAACCTTCCAGCGCAAGTCATGGACATCATCGACAGCGCGCTGCGCTCCATCCCCGTCCTCGGCG | Extend Pr∆*lysA* |
| PrExtender∆*lysA*rv | CCCCTGGTCCGAGGCCGACGTATTGGCCGTCGATGCGCATCAGTGGCCCAACAGTTCTGCTACCGCGTCCACGA | Extend Pr∆*lysA* |
| Pr*lysA*_TGA_HindIIIfwd | CCACGAAAGATCAAGTCGCCCAAATCACCATCGCCTGAAGCTTCAAGGCGCGCGGCTACACCCGCAGCGAATG | Ms6 *lysA*_TGA_ |
| Pr*lysA*_TGA_HindIIIrv | CATTCGCTGCGGGTGTAGCCGCGCGCCTTGAAGCTTCAGGCGATGGTGATTTGGGCGACTTGATCTTTCGTGG | Ms6 *lysA*_TGA_ |
| Pr*lysA*_GTG→TGG_MscIfwd | ATACCTCGACAAGTACTGGCCCGCCGATGGAGGTACCGCCTGGCCAGACGAACCACGCCCCGACTTCAACGAGTTTCCGATCTGGT | Ms6 *lysA*_GTG→TGG_ |
| Prl*ysA*_GTG→TGG_MscIrv | ACCAGATCGGAAACTCGTTGAAGTCGGGGCGTGGTTCGTCTGGCCAGGCGGTACCTCCATCGGCGGGCCAGTACTTGTCGAGGTAT | Ms6 *lysA*_GTG→TGG_ |
| Pr*lysA*His_6_tag | AACCCTCGTGGACGCGGTAGCAGAACTGTTGGGCCACCACCACCACCACCACTGATGCGCATCGACGGCCAATACGTCGGCCTCGGACC | Ms6 *lysA*His_6_tag insertion |
| PrExt*lysA*His_6_tagfwd | CGAGATCCTGCGGCAACTGCGCGGATACAACCTCACTGGCTGGCCGCAGCTCGGCGGAAAAACCCTCGTGGACGCGGTAGCAGAACTGTT | ExtendPr*lysA*His_6_tag |
| PrExt*lysA*His_6_tagrv | TAGGAGAACTTGCGCCGCATGAACGCTTTGATCTTGCGGATCTCGTCGGATCTGTCCCCTGGTCCGAGGCCGACGTATTGGCCGTCGATG | ExtendPr*lysA*His_6_tag |
| *lysA*His_6_tagfwd | GAACTGTTGGGCCACCACCACCACCAC | Ms6 *lysA*His_6_tag^TAG^ |
| *lysB*rv | GATACCCCATGACGTACTTGGTTTCGGCG | Ms6 *lysA*His_6_tag ^FP^ |
| PrP1fwd | CGGTACTAGTCGGCCTCGGCCTGC | Ms6 *lysA*_TGA_ ^FP^ |
| Pr*lysA*180bpr | GCAAGCTTGTGTGGGTAGGAGCCGTCC | Ms6 *lysA*_TGA_ ^FP^ |
| gp2B | CGATGCTGCAGTCAGTGGCCCAACAC | Ms6 *lysA*_GTG→TGG_ ^FP^ |
| Δ*lysA*DADA-PCR | GAATCCTCGGGAGCAAACGGTGACCTGG | Ms6 Δ*lysA*^DP^ |
| *lysB*DADA-PCR | GATACCCCATGAACGTACTTGGTTTCGGCG | Ms6 Δ*lysA*^DP^ |

**^a^** underlined bases were added to provide additional restriction sites

**^CP^** Cloning Primer

**^FP^**Flanking primer.

**^DP^** DADA-PCR primer

**^TAG^**Primer specific to the tag sequence.
